# Evaluation of Sodium Hypochlorite Irrigant, Bingpeng Irrigant, and Fufang Bingpeng Irrigant as Endodontic Irrigants During Passive Ultrasonic Irrigation

**DOI:** 10.3389/fcimb.2019.00145

**Published:** 2019-05-10

**Authors:** Yan Shi, Zhipeng Deng, Yulu Yang, Lanyue Cui, Tingtao Chen, Mingjing Hu, Lei Xie, Jian Yang

**Affiliations:** ^1^Department of Conservative Dentistry and Endodontics, Stomatology Hospital of Nanchang University, Jiangxi Provincial Key Laboratory of Oral Biology, Nanchang, China; ^2^Stomatology School of Nanchang University, Nanchang, China; ^3^Institute of Translational Medicine, Nanchang University, Nanchang, China

**Keywords:** root canal irrigant, NaClO irrigant, Fufang Bingpeng irrigant, cytotoxicity, cleaning efficacy, high-throughput sequencing

## Abstract

Given the increasing prevalence of antibiotic resistance among bacterial strains and the side effects caused by synthetic drugs, it is increasingly important to investigate potential herbal alternatives. In the present study, antimicrobial, cell cytotoxicity, and cleaning tests were performed to evaluate the potential of Fufang Bingpeng irrigant as a root canal irrigant, in addition to q-PCR and high-throughput sequencing analyses. Our *in vitro* results showed a low minimum inhibitory concentration (MIC) and minimum bactericidal concentration (MBC) of Fufang Bingpeng irrigant against *Porphyromonas gingivalis* ATCC 33277 (6.25 and 12.5%, respectively), *Prevotella intermedius* ATCC 25611 (6.25 and 6.25%, respectively), *Fusobacterium nucleatum* ATCC 25286 (6.25 and 6.25%, respectively), *Enterococcus faecalis* ATCC 19433 (25 and 25%, respectively), and *Bacteriodes fragilis* ATCC 25285 (12.5 and 12.5%, respectively). Furthermore, it effectively removed the remaining debris and increased the number of open dentinal tubules in root canals compared to the NaCl irrigant (*p* < 0.05). Fufang Bingpeng irrigant also presented low cytotoxicity to L929 cells compared to the NaClO irrigant. The *in vivo* results indicated that all irrigants used significantly reduced the number of bacteria compared to the number prior to treatment, and only 1/104.95 bacteria remained in the root canal following the use of Fufang Bingpeng irrigant (*p* < 0.001). Moreover, the high-throughput sequencing results indicated that all irrigants markedly enhanced the α diversity in the root canal compared to the before preparation control group, while Fufang Bingpeng maintained better microbial diversity than other groups. Therefore, Fufang Bingpeng irrigant presents a promising alternative for use as a root canal irrigant in clinical settings.

## Introduction

The bacteria present in the root canal play an important role in the pathologies of pulpitis and periapical periodontitis, and the success of root canal therapy is mainly dependent on controlling bacterial infection inside the root canal (Rödig et al., 2010). Root canal irrigation represents one of the basic steps for root canal therapy, which can effectively control the bacterial infection inside the root canal (Vouzara et al., 2016).

Currently, NaClO irrigation is commonly used in the clinic due to its sound antibacterial properties, low cost and long period of action, while its shortcomings (e.g., high toxicity, unpleasant smell, potential corrosiveness, and allergy) hinder its use (Sabins et al., 2003; Rödig et al., 2010). Other irrigants, such as chlorhexidine and ardenia jasmine gargle, have also been used in the clinic; however, they have several drawbacks (antimicrobial-resistant strains and side effects), thus it is necessary to find more suitable irrigants (Stuart et al., 2006; Prabhakar et al., 2010; Sibley et al., 2012).

In the present study, Fufang Bingpeng irrigant, made by pure Chinese Medicines, was investigated by our group. Specifically, we assessed its effect on obligate anaerobic microorganisms, remaining debris and open dentinal tubules in the apical and middle thirds of the root canal, as well as the growth of L929 cells and microbial diversity in the root canal.

## Materials and Methods

### The Minimum Inhibitory Concentration (MIC) and Minimum Bactericidal Concentration (MBC) of Irrigants

The pathogens *Porphyromonas gingivalis* ATCC 33277, *Prevotella intermedius* ATCC 25611, *Fusobacterium nucleatum* ATCC 25286, *Enterococcus faecalis* ATCC 19433, and *Bacteriodes fragilis* ATCC 25285 were cultured in brain/heart infusion (BHI) medium for 24 h in an incubator with an atmosphere of 80% N_2_, 10% CO_2_, and 10% H_2_. Then, Fufang Bingpeng irrigant and Bingpeng irrigant were added to the BHI plate to make final concentrations of 50, 25, 12.5, 6.25, 3.1, 1.6, 0.8, 0.4, 0.2, and 0.1%; and NaClO irrigant was added at 4, 2, 1, 0.5, 0.25, 0.125, 0.062, 0.031, 0.016, and 0.008%. Pathogens were then inoculated onto the plates, and their MIC and MBC were determined using the viable cell counting method (Jiang et al., 2016).

### Cytotoxicity of Tested Irrigants

L929 mouse fibroblasts cells were maintained in DMEM supplemented with 15% FBS at 37°C in a 5% CO_2_ incubator. Different concentrations of irrigants were dissolved in cell culture media, and their effect on the viability of L929 cells was determined by MTT assay (Hu et al., 2018).

L929 cells were plated at a density of 10,000 cells per well in 100 μl of complete culture media. Cells were then treated with designated concentrations of NaClO irrigant (0.1%, 0.5%, 2.5% and 5%), Bingpeng irrigant (self-made; 6.25, 12.5, 25, and 50%) and Fufang Bingpeng irrigant (self-made; 6.25, 12.5, 25, and 50%) in 96-well microtiter plates for 30 min, 1 h, 6 h and 12 h at 37°C in a humidified incubator. After incubation until a specific stage, MTT reagent (20 μl, 5 mg/mL) was added and incubated for 3.5 h, then the MTT solution was carefully removed and 150 μl of DMSO was added. The absorbance was recorded on a microplate reader at a wavelength of 570 nm. Corresponding volumes of NaCl irrigant were used as the control.

### Evaluation of the Cleaning Efficacy of the Tested Irrigants

Forty bicuspid teeth were collected from children (aged 11–13 years) undergoing orthodontic extraction. These children received no pulp canal therapy, and their apical foramen was completely developed and without caries. The collected teeth were then randomly divided into four groups and stored in physiological saline for further use.

Root canal preparation was performed using gradually deepening preparatory technology with a K-file. Between each instrument, the canals were irrigated with 5 ml NaCl irrigant (0.9%), NaClO irrigant (0.5% NaClO + 17% EDTA), Bingpeng irrigant (100%) or Fufang Bingpeng irrigant (100%), then 3 ml of physiological saline was used to terminate the effect of different irrigants on the root canal. The root canal was dried with paper points, and the root canal orifice was sealed with a cotton pellet and Cavit. A single operator with experience in this method and instrumentation prepared all canals.

With the aid of a surgical operating microscope, a longitudinal groove was cut in the root using the end of a diamond bur. The roots were removed from the crown and then split by placing a surgical blade in the groove and striking the blade with a small mallet. Images of the split roots were made using a digital camera set at 1:1. The images were transferred to a computer with Adobe Photoshop CS software and enlarged to 100 × the original size. Lines were superimposed over the canals at 0, 3, and 6 mm from the apical constriction. The debris in each canal was traced, and the total number of pixels occupied by debris was determined using the histogram function in the software. The outline of the canal was then traced, and the same feature of the software reported the total pixels occupied by the canal. The percentage of debris was calculated by dividing the number of debris pixels by the total number of pixels, representing the entire area of the canal. The percentage of debris was calculated for the apical third and middle third of each canal.

Six specimens were randomly selected from each group. The teeth were split and their two halves were mounted in specimen holders. They were then placed in a vacuum chamber and coated with a 20-nm-thick gold-palladium layer for scanning electron microscopy (Hu et al., 2018). The apical and middle thirds of each root canal were selected, and photomicrographs of the central part were taken at 500 × magnification on the longitudinal plane and then enlarged to 110 × 120 mm. A square 72-mm grid composed of 64 equal squares, of which 16 were randomly selected, was superimposed onto the scanning electron microscope (SEM) photomicrograph. The number of open dentinal tubules inside each square were counted by a single observer. The sum of the 16 squares of each grid was calculated, obtaining the total number of open dentinal tubules.

### Extraction of Bacterial DNA

Between January 2018 and July 2018, 40 patients with periapical periodontitis were selected. Root canal preparation was performed using gradually deepening preparatory technology with K-files and S3 NiTi files. Between each instrument, the canals were irrigated with either 5 ml NaCl irrigant (*n* = 10), NaClO irrigant (*n* = 10), Bingpeng irrigant (*n* = 10), or Fufang Bingpeng irrigant (*n* = 10). Ultrasonic irrigation was performed for 10–20 s, followed by ultrasonic irrigation with 3 ml EDTA for 10–20 s. Physiological saline (5 ml) was then used to terminate the effect of the different irrigants on the root canal. Samples were collected before and after the preparation for further analysis.

All human tests were approved by the Committee on the Ethics of Human Experiments of Stomatology Hospital of Nanchang University (Jiangxi, China). Patient samples were obtained with written informed consent in accordance with the requirements of the Ethics Committee.

### Real-Time PCR

The number of bacteria in the root canal samples were determined by real-time PCR using the ABI 7900HT fast real-time PCR system (Applied Biosystems, USA). The reaction buffer consisted of primers (338F, 5′-ACTCCTACGGGAGGCAGCAG-3′; 518R, 5′- ATTACCGCGGCTGCTGG-3′), ROX reference dye and SYBR® Primer EX Taq II (TaKaRa), and amplification began at 94°C for 8 min. The temperatures used for degeneration, annealing and extension were 94, 58, and 71°C, respectively. The relative expression levels of the target bacteria were analyzed using the 2^−ΔΔ^Ct method (Chen et al., 2018).

### High-Throughput Sequencing

Primers (515F, 5′-GTG CCA GCM GCC GCG GTA A-3′; and 806R, 5′-GGA CTA CVS GGG TAT CTA AT-3′) were used to amplify the V4 region of the 16S rRNA genes of the extracted genomic DNA (GenBank accession number PRJNA496554) (Xin et al., 2016). FLASH was used to merge overlapped tags, the UPARSE software package was used to analyze sequences, and in-house Perl scripts were used to analyze the alpha (within samples) and beta (among samples) diversity. Sequences with high similarity (≥97%) were considered the same OTUs, and the QIIME software package was applied to analyze the weighted UniFrac distance.

### Data Analysis

Data are presented as the mean ± standard deviation (SD), and Prism software version 7.0 (GraphPad Software, San Diego, CA, USA) was used to perform the statistical analyses. Error probabilities of *p* < 0.05 were considered statistically significant.

## Results

### Antibacterial Effect of Irrigants

The MIC and MBC of the tested irrigants on *P. gingivalis* ATCC 33277, *P. intermedius* ATCC 25611, *F. nucleatum* ATCC 25286, *E. faecalis* ATCC 19433, and *B. fragilis* ATCC 25285 are shown in Table 1. All tested irrigants showed a significant antibacterial effect, and the NaClO irrigant possessed the lowest MIC and MBC values for *P. gingivalis* ATCC 33277 (0.035 and 0.035%, respectively), *P. intermedius* ATCC 25611 (0.070 and 0.070%, respectively), *F. nucleatum* ATCC 25286 (0.018 and 0.018%, respectively), *E. faecalis* ATCC 19433 (0.28 and 0.28%, respectively) and *B. fragilis* ATCC 25285 (0.070 and 0.070%, respectively). Although the Fufang Bingpeng irrigant possessed a weaker effect on pathogens, its antibacterial effect was stronger than the Bingpeng and NaCl irrigants.

**Table 1:** MIC and MBC of NaClO irrigant, Bingpeng irrigant, and Fufang Bingpeng irrigant against obligated anaerobic microorganisms.

**Drugs**	***P. gingivalis* ATCC 33277**	***P. intermedius* ATCC25611**	***F. nucleatum* ATCC 25286**	***E. faecalis* ATCC 19433**	***B. fragilis* ATCC25285**
**CONCENTRATION OF NACLO IRRIGANT (%)**					
MIC	0.035	0.070	0.018	0.28	0.07
MBC	0.035	0.070	0.018	0.28	0.07
**CONCENTRATION OF BINGPENG IRRIGANT (%)**					
MIC	12.5	12.5	12.5	50	25
MBC	25	12.5	25	>50	25
**CONCENTRATION OF FUFANG BINGPENG IRRIGANT (%)**					
MIC	6.25	6.25	6.25	25	12.5
MBC	12.5	6.25	6.25	25	12.5

### Cytotoxicity of Irrigants on L929 Cells

**Figure 1:**
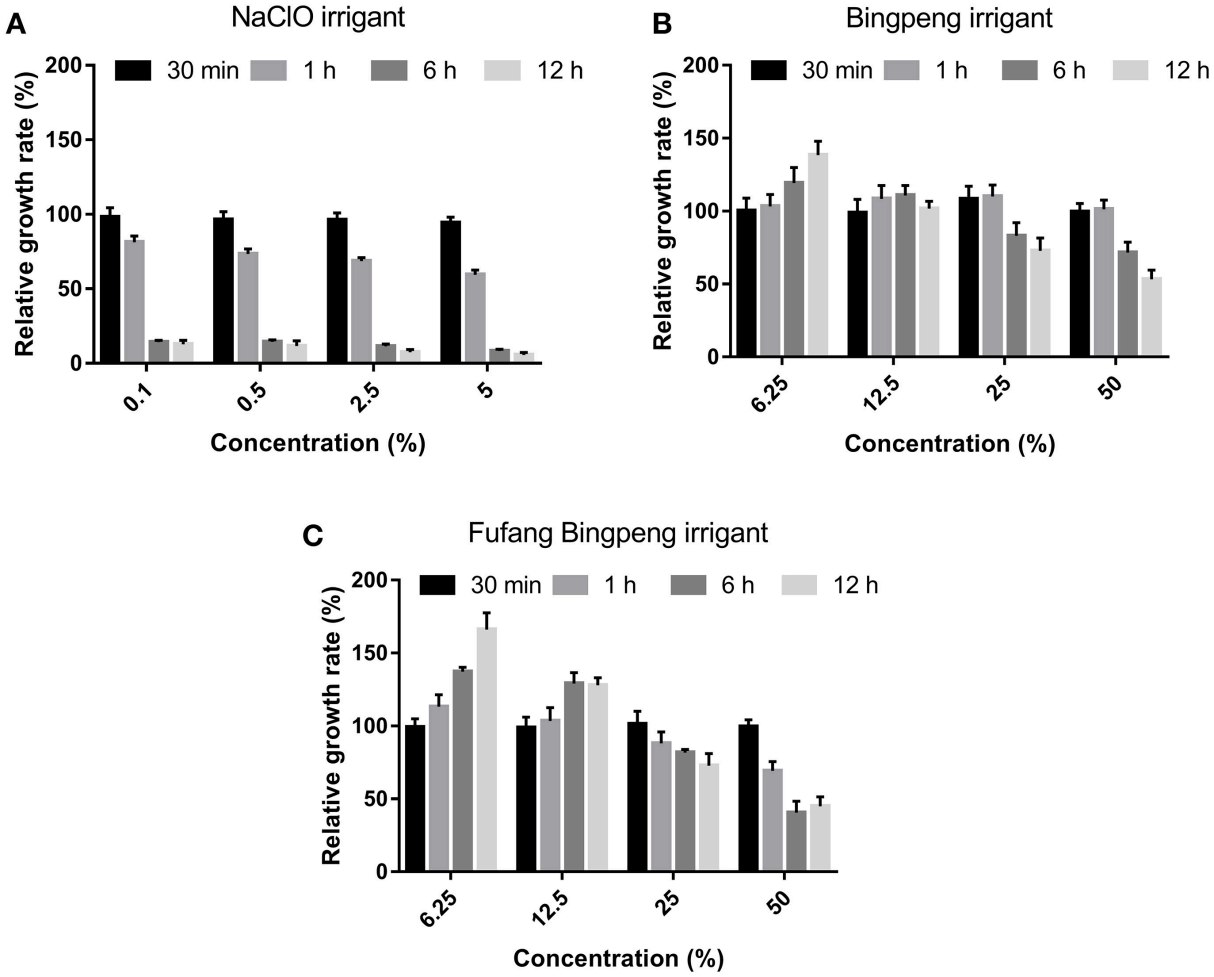
Effect of different concentrations of NaClO irrigant **(A)**, Bingpeng irrigant **(B)** and Fufang Bingpeng irrigant **(C)** on the relative growth rate of L929 cells compared to the NaCl irrigant group.

As shown in Figure 1, dose-dependent and time-dependent effects were observed when the cytotoxicity of irrigants was tested. After 30 min, all irrigants showed little cytotoxicity on L929 cells at all tested concentrations. After 1 h, the NaClO irrigant showed strong cytotoxicity, especially at concentrations >0.5%. At 6 and 12 h, few viable cells were obtained at all tested concentrations. For the Fufang Bingpeng irrigant and the Bingpeng irrigant, even low concentrations (6.25 and 12.5%, respectively) showed a growth-promoting effect, while high concentrations (25 and 50%, respectively) showed a serious cytotoxic effect on L929 cells, with the Fufang Bingpeng irrigant possessing higher cytotoxicity than the Bingpeng irrigant.

### Effect of Irrigants on Cleaning Efficacy

**Figure 2:**
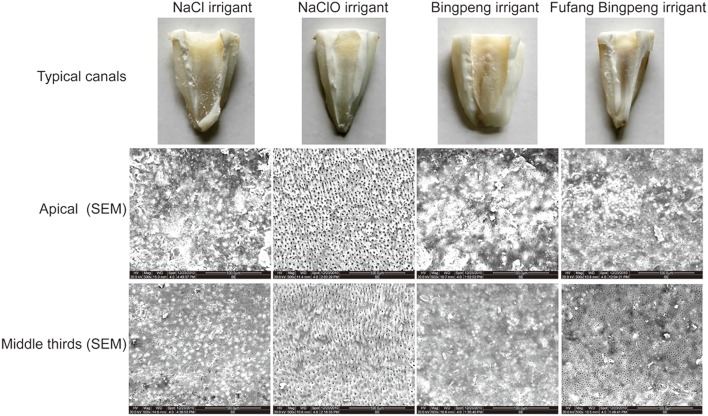
Typical canals with debris and SEM (500*) of the apical and middle thirds of root canals treated with the NaCl irrigant, NaClO irrigant, Bingpeng irrigant, and Fufang Bingpeng irrigant.

Overall, the amount of debris in the NaCl irrigant group was markedly higher than other groups (*P* < 0.05), and no significant differences were observed among the NaClO, Fufang Bingpeng and Bingpeng irrigant groups in the apical part of the root canal (Figure 2 and Table 2). Similarly, debris in the middle third of the root canal in the NaCl irrigant group was also higher than that in the NaClO irrigant group. Moreover, the SEM results indicated that there were fewer open dentinal tubules in the NaCl irrigant group than the other three groups (*p* < 0.05, apical part of the root canals), and the numbers in the middle third of the root canal were markedly lower than those in the NaClO and Fufang Bingpeng irrigant groups (Figure 2 and Table 2). In summary, the cleaning efficacy of irrigants were as follows: NaClO irrigant>Fufang Bingpeng irrigant>Bingpeng irrigant>NaCl irrigant.

**Table 2:** Effect of NaCl irrigant, NaClO irrigant, Bingpeng irrigant, and Fufang Bingpeng irrigant on remaining debris and open dentinal tubules in the apical and middle thirds of the root canals.

**Drugs**	**Remaining debris (%**, ***n*** **=** **10)**		**Open dentinal tubule number (*****n*** **=** **6)**	
	**Apical thirds**	**Middle thirds**	**Apical thirds**	**Middle thirds**
NaCl irrigant	20.02 + 6.02	12.41 + 5.61	9.83 + 7.86	13.33 + 5.24
NaClO irrigant	6.65 + 4.81^*^	4.55 + 6.02^*^	70.17 + 37.63^*^	85 + 26.33^*^
Bingpeng irrigant	10.11 + 6.03^*^	7.07 + 3.01^*^	40.03 + 16.43^*^	70.5 + 25.38^*^
Fufang Bingpeng irrigant	9.63 + 6.43^*^	6.86 + 2.81^*^	45.33 + 18.45	75.5 + 34.58^*^

*x+s*,

* *p < 0.05 compared with NaCl irrigant*.

### Effects of Irrigants on the Relative Bacterial Number in the Root Canal

**Figure 3:**
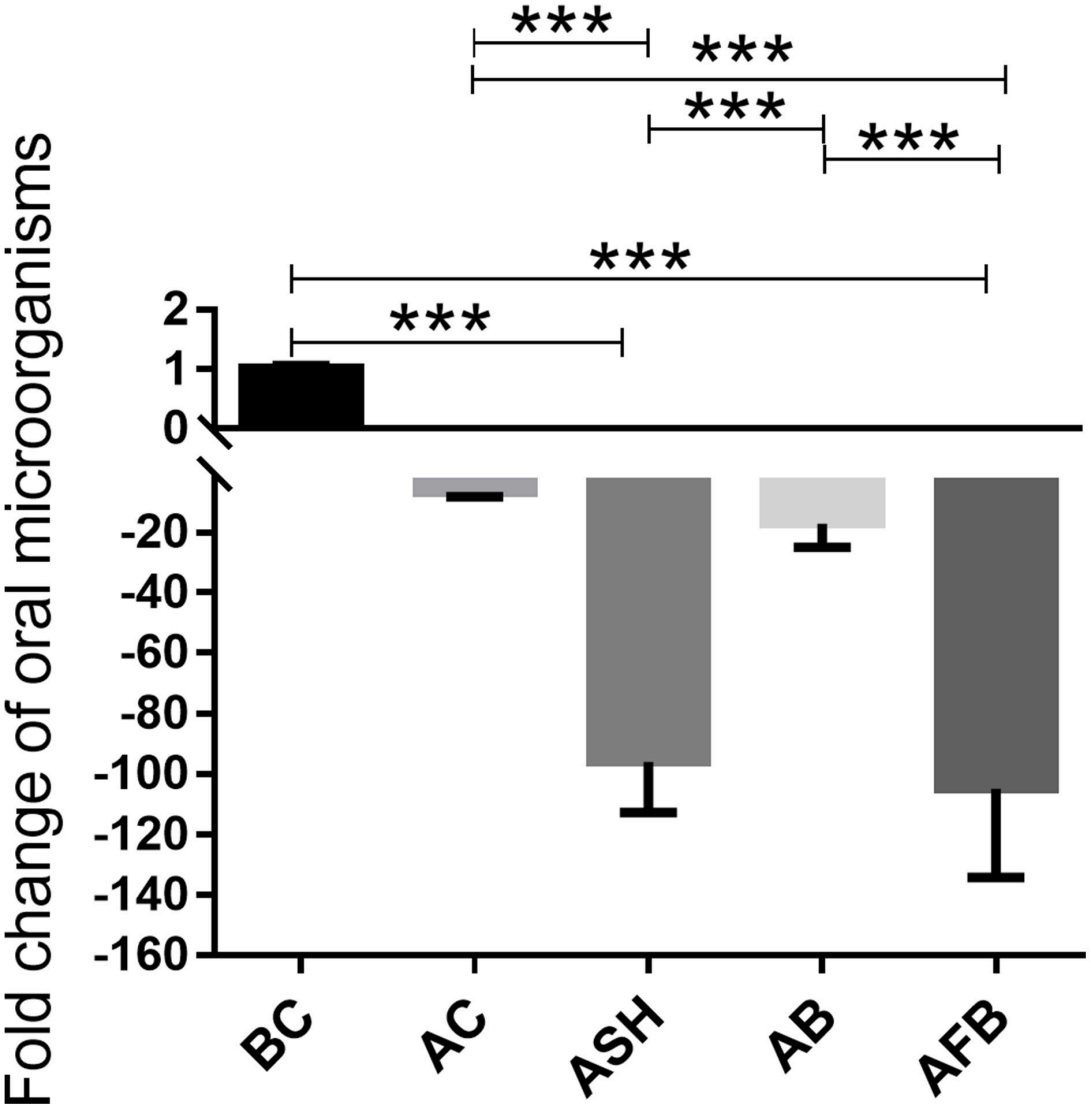
Effects of the NaCl irrigant, NaClO irrigant, Bingpeng irrigant and Fufang Bingpeng irrigant on the elimination of bacteria in the oral cavity, assessed using q-PCR. BC, microbiota in the oral cavity of patients before treatment; AC, microbiota in the oral cavity of patients after treatment with the NaCl irrigant; ASH, microbiota in the oral cavity of patients after treatment with the NaClO irrigant; AB, microbiota in the oral cavity of patients after treatment with the Bingpeng irrigant; AFB, microbiota in the oral cavity of patients after treatment with the Fufang Bingpeng irrigant. ***p < 0.001.

Finally, we evaluated the effects of irrigants on bacterial diversity in the root canal. As shown in Figure 3, the NaCl, NaClO, Bingpeng, and Fufang Bingpeng irrigants all significantly reduced the number of bacteria compared to the number prior to treatment (p < 0.001). Compared to the bacterial number before the preparation, 99.59-fold and 104.95-fold reductions in bacterial numbers were obtained in the NaClO and Fufang Bingpeng irrigant groups, respectively.

### Effects of Irrigants on the Microbial Composition of the Root Canal

High-throughput sequencing was used to explore the effects of the NaCl, NaClO, Bingpeng, and Fufang Bingpeng irrigants on the microbial composition of the root canal. In total, 3,547,824 filtered clean tags (73,913 tags/sample) and 44,834 OTUs were obtained from all samples, with an average of 934.04 OTUs per group (Table S1).

**Figure 4:**
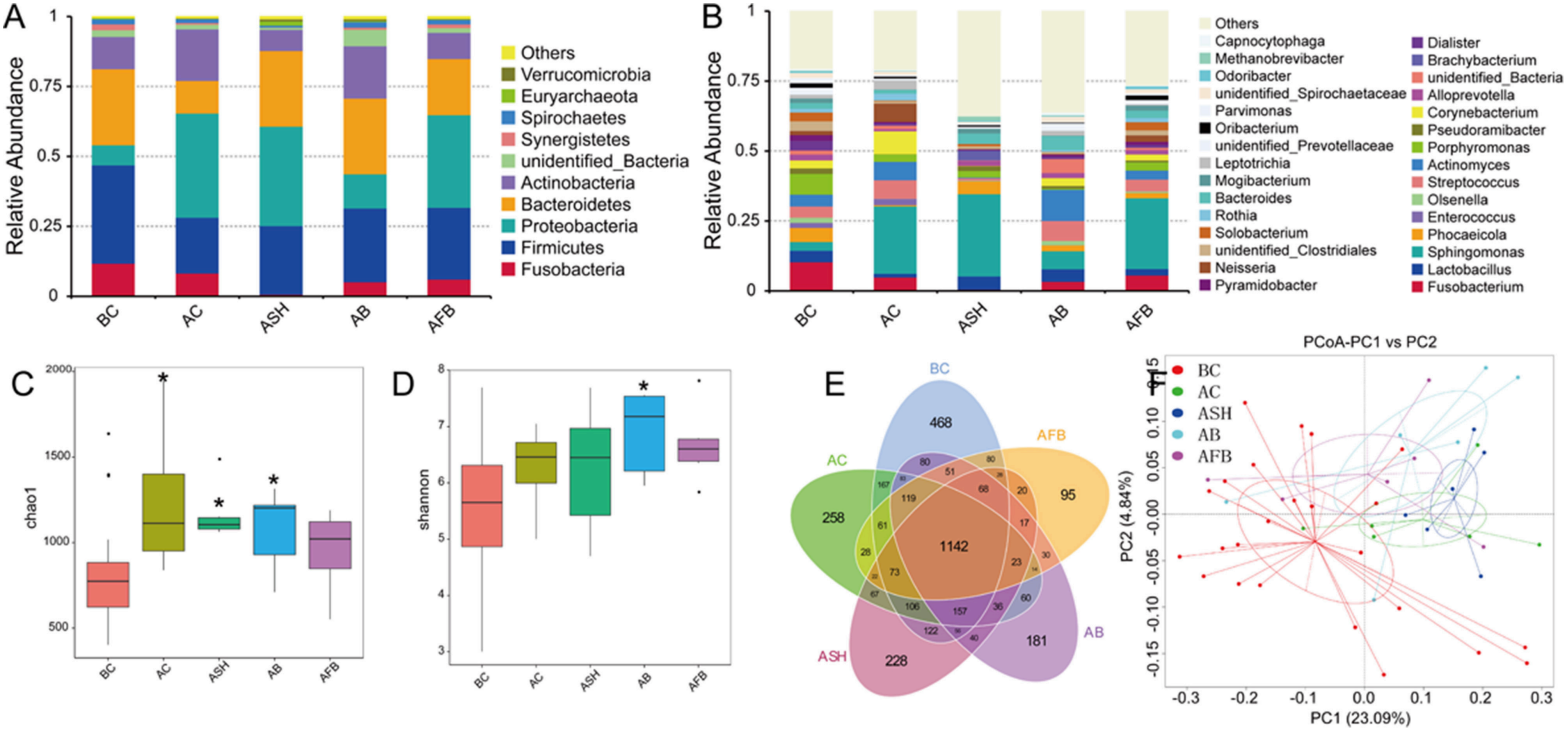
Evaluation of the effects of the NaCl irrigant, NaClO irrigant, Bingpeng irrigant and Fufang Bingpeng irrigant on the oral microbiota. **(A)** Relative bacterial abundance at the phylum level, **(B)** relative bacterial abundance at the genus level, **(C)** the Chao1 index, **(D)** the Shannon index, **(E)** Scalar-Venn representation of the vaginal microbiota, and **(F)** principal coordinates analysis (PCoA). BC, microbiota in the oral cavity of patients before treatment; AC, microbiota in the oral cavity of patients after treatment with the NaCl irrigant; ASH, microbiota in the oral cavity of patients after treatment with the NaClO irrigant; AB, microbiota in the oral cavity of patients after treatment with the Bingpeng irrigant; AFB, microbiota in the oral cavity of patients after treatment with the Fufang Bingpeng irrigant. *p < 0.05 compared to the BC group.

**Figure 5:**
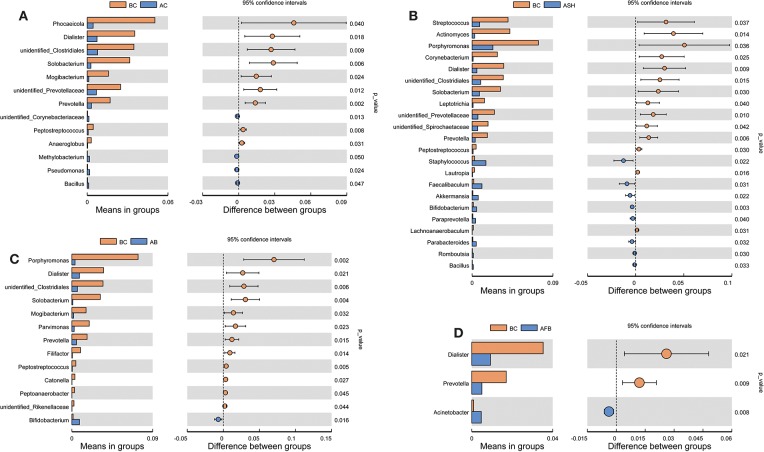
Analysis of species differences among BC vs. AC **(A)**, BC vs. ASH **(B)**, BC vs. AB **(C)** and BC vs. AFB **(D)** groups. BC, microbiota in the oral cavity of patients before treatment; AC, microbiota in the oral cavity of patients after treatment with the NaCl irrigant; ASH, microbiota in the oral cavity of patients after treatment with the NaClO irrigant; AB, microbiota in the oral cavity of patients after treatment with the Bingpeng irrigant; AFB, microbiota in the oral cavity of patients after treatment with the Fufang Bingpeng irrigant.

As shown in Figure 4, the irrigants used in the present study had an obvious effect on the microbial composition at both the phyla and genus levels, and the Fufang Bingpeng irrigant resulted in a marked reduction in *Fusobacterium, Enterococcus* and *Pseudoramibacter* at the genus level (Figures 4A,B). In addition, the Chao1 index and Shannon index indicated that all irrigants markedly enhanced the α diversity in the root canal compared to the before preparation control group (BC), especially for the NaCl irrigant, NaClO irrigant and Bingpeng irrigant groups (*p* < 0.01). When analyzed using the Venn method, 1142 core OTUs were found in all groups, with 468, 258, 228, 181, and 95 OTUs found in the BC group, the after preparation control group (AC), the after preparation NaClO irrigant group (ASH), the after preparation Bingpeng irrigant group (AB) and the after preparation Fufang Bingpeng irrigant group (AFB), respectively (Figure 4E). The principal coordinates analysis (PCoA) indicated that although irrigants changed the microbial diversity compared to the BC group, the AFB possessed the most similar bacterial composition to the BC group (Figure 4F). When the significant changes in bacteria were compared between the BC vs. AC, BC vs. ASH, BC vs. AB and BC vs. AFB groups, we found that *Prevotella* in the AC, ASF, AB, and AFB groups were significantly reduced compared to the BC group (Figure 5).

## Discussion

Oral microorganisms, especially opportunistic pathogens, cause primary endodontic infections due to a gradual increase in number over long periods in the root canal (Prabhakar et al., 2010). As an adjunct, various irrigants have been applied to improve antimicrobial cleaning and shaping in endodontics and to eliminate most of the bacteria. However, studies indicate that some bacteria can survive in the root canal (Gomes et al., 2010). The NaOCl irrigant is widely used, representing the most common irrigant used during endodontic treatment. However, its high toxicity, unpleasant taste and inability to dissolve dentin debris have hindered its use (Stuart et al., 2006; Da et al., 2008). Therefore, it is necessary to find a suitable irrigant with low toxicity and high efficiency.

The Bingpeng irrigant originates from “Bingpeng powder,” which contains borneol, borax, cinnabar and weathered sodium sulfate, and has been suggested to effectively inhibit bacterial growth and eliminate inflammation (Shi et al., 2011; Wang, 2011). To enhance its antimicrobial effect and cleaning efficiency and decrease its toxicity, cinnabar was removed from the Bingpeng irrigant, and *Angelica archanglica* and *Asurum uropeum* were added. The resulting “Fufang Bingpeng” irrigant has greatly enhanced antibacterial and anti-inflammatory effects, and has an additional use for treating detumescence and abscesses, and as a pain reliever (Shi et al., 2011).

Anaerobic bacteria usually persist in treated root canals and are often resistant to traditional antibiotics. When anaerobic bacteria grow as a biofilm, antimicrobial agents cannot enter. Furthermore, a 1500-fold increase in the rate of antibiotic resistance had been observed compared to planktonic cells (Gordon et al., 1988; Mah and O'toole, 2001). As shown in Table 1, the NaClO irrigant effectively inhibited all tested pathogens and showed the lowest MIC and MBC values. Although the antibacterial effect of the Fufang Bingpeng irrigant was inferior to the NaClO irrigant, its MIC and MBC for *P. gingivalis* ATCC 33277, *P. intermedius* ATCC 25611, *F. nucleatum* ATCC 25286, *E. faecalis* ATCC 19433, and *B. fragilis* ATCC 25285 were as low as 6.25 vs. 12.5%, 6.25 vs. 6.25%, 6.25 vs. 6.25%, 25 vs. 25%, and 12.5% vs. 12.5%, respectively. The NaClO and EDTA irrigants showed a better effect on the amount of remaining debris and number of open dentinal tubules in the apical and middle thirds of the root canal (Table 2), while NaClO markedly inhibited the growth of L929 cells, even at low concentrations (Figure 1). The residual debris and smear layer in infected root canals harbor various microorganisms and their byproducts, which also hinders the ability of the irrigants to directly contact the entire root canal wall (Zehnder, 2006). The Fufang Bingpeng irrigant resulted in better removal of the smear layer, pulp tissue and dentin debris, and even enhanced the growth of L929 cells at low concentrations (Figure 1 and Table 2).

The oral cavity contains various microorganisms that are associated with oral diseases. Bacterial infections within the root canal are usually polymicrobial in nature, and it is rare that a single species causes oral disease (Chen et al., 2017). Therefore, q-PCR and high-throughput sequencing were used to evaluate the effects of the NaCl, NaClO, Bingpeng, and Fufang Bingpeng irrigants on bacterial diversity in the root canal. As shown in Figure 3, all tested irrigants effectively reduced the bacterial number compared to the BC group, and 99.59- and 104.95-fold reductions were observed in the NaClO irrigant group and Fufang Bingpeng irrigant group, respectively (Figures 3, 5). Moreover, the results of the high-throughput sequencing analysis indicated that although all tested irrigants greatly reduced the bacterial number (Figure 3) and changed the microbial composition compared with the BC group at the phyla and genus levels (Figures 4A,B), the α diversity analysis clearly showed that all tested irrigants enhanced the bacterial abundance (Figures 4C,D). Most importantly, both the α diversity analysis and PCoA results indicate that the Fufang Bingpeng irrigant could better sustain the microbial diversity in the BC and AC groups, and the similar bacterial diversity with formal ones will help improve oral health (Figure 4).

In the present study, the Fufang Bingpeng irrigant showed a sound antimicrobial effect, low cytotoxicity and high cleaning efficacy *in vitro*, and the *in vivo* results showed that the Fufang Bingpeng irrigant significantly reduced the bacterial number and sustained the microbial diversity, which helps to avoid oral infection and protect oral health. Therefore, the use of Fufang Bingpeng irrigant as a root canal irrigant might prove to be advantageous considering its desirable characteristics compared to the NaClO irrigant. However, as all PCR based technologies cannot differentiate viable or dead cells, the PMA-modified viability PCR technology should be introduced in our further work.

## Author Contributions

YS, TC, and JY designed the experiment. ZD, YY, LC, MH, and LX performed the experiments. TC and YS analyzed the data and wrote the manuscript. All authors discussed the results and commented on the manuscript.

### Conflict of Interest Statement

The authors declare that the research was conducted in the absence of any commercial or financial relationships that could be construed as a potential conflict of interest.
