# Supplementary material for: Evaluation of Sodium Hypochlorite Irrigant, Bingpeng Irrigant, and Fufang Bingpeng Irrigant as Endodontic Irrigants During Passive Ultrasonic Irrigation
Source: Front Cell Infect Microbiol. 2019 May 10;9:145. doi: 10.3389/fcimb.2019.00145 (PMC6522938; doi:10.3389/fcimb.2019.00145)
Supplement: Supplementary file 1 [file Table_1.docx]

**Supplementary Material**

**Table S1** Number of raw reads, clean reads, AvgLen, Unique Tag, OUT number and effective of fecal micriobiota in groups BC, AC, ASH, AB and AFB by high‑throughput sequencing. BC, the microbiota in oral cavity of patients before treatment; AC, the microbiota in oral cavity of patients after treatment with NaCl irrigant; ASH, the microbiota in oral cavity of patients after treatment with NaClO irrigant; AB, the microbiota in oral cavity of patients after treatment with Bingpeng irrigant; AFB, the microbiota in oral cavity of patients after treatment with Fufang Bingpeng irrigant.

| Sample Name | Raw reads | Clean Reads | AvgLen (nt) | Unique Tag | OUT number | Effective (%) |
| --- | --- | --- | --- | --- | --- | --- |
| BC1 | 62905 | 59815 | 252 | 3006 | 958 | 95.09 |
| BC2 | 58914 | 57717 | 252 | 5066 | 1380 | 97.97 |
| BC3 | 86742 | 82668 | 252 | 2721 | 588 | 95.3 |
| BC4 | 76589 | 73635 | 251 | 2066 | 713 | 96.14 |
| BC5 | 63131 | 57016 | 252 | 1799 | 439 | 90.31 |
| BC6 | 91299 | 86328 | 252 | 4439 | 727 | 94.55 |
| BC7 | 81787 | 80162 | 251 | 7449 | 710 | 98.01 |
| BC8 | 77948 | 74136 | 252 | 2227 | 627 | 95.11 |
| BC9 | 84500 | 80133 | 250 | 2235 | 583 | 94.83 |
| BC10 | 82743 | 76594 | 251 | 1684 | 484 | 92.57 |
| BC11 | 84106 | 80188 | 252 | 2772 | 953 | 95.34 |
| BC12 | 90938 | 81873 | 251 | 4497 | 548 | 90.03 |
| BC13 | 70506 | 68351 | 252 | 3907 | 840 | 96.94 |
| BC14 | 89767 | 86163 | 253 | 6517 | 1327 | 95.98 |
| BC15 | 88247 | 80181 | 251 | 4001 | 1547 | 90.86 |
| BC16 | 62612 | 59175 | 252 | 2112 | 868 | 94.51 |
| BC17 | 83582 | 80925 | 251 | 8334 | 479 | 96.82 |
| BC18 | 85772 | 79468 | 252 | 6173 | 762 | 92.65 |
| BC19 | 62369 | 56699 | 254 | 9626 | 536 | 90.91 |
| BC20 | 82400 | 80123 | 252 | 7337 | 807 | 97.24 |
| BC21 | 82104 | 80149 | 252 | 5969 | 819 | 97.62 |
| BC22 | 80684 | 78057 | 252 | 7845 | 672 | 96.74 |
| BC23 | 82061 | 75294 | 252 | 7967 | 901 | 91.75 |
| BC24 | 83997 | 80238 | 252 | 2778 | 512 | 95.52 |
| AC1 | 82547 | 80238 | 252 | 4659 | 797 | 97.2 |
| AC2 | 75039 | 72850 | 252 | 2106 | 981 | 97.08 |
| AC3 | 86701 | 80119 | 252 | 6863 | 1851 | 92.41 |
| AC4 | 82812 | 80287 | 251 | 3616 | 1345 | 96.95 |
| AC5 | 76259 | 73074 | 252 | 5069 | 961 | 95.82 |
| AC6 | 85335 | 77172 | 252 | 4692 | 1199 | 90.43 |
| ASH1 | 85960 | 80050 | 252 | 3347 | 1056 | 93.12 |
| ASH2 | 85407 | 80282 | 252 | 3768 | 974 | 94 |
| ASH3 | 75076 | 70308 | 252 | 3272 | 1084 | 93.65 |
| ASH4 | 65862 | 61269 | 252 | 2721 | 1091 | 93.02 |
| ASH5 | 82325 | 80136 | 251 | 4158 | 1130 | 97.34 |
| ASH6 | 84772 | 80207 | 251 | 2804 | 1355 | 94.61 |
| AB1 | 75547 | 73859 | 252 | 3993 | 1351 | 97.76 |
| AB2 | 82149 | 80097 | 252 | 7700 | 1098 | 97.5 |
| AB3 | 70827 | 66390 | 252 | 8361 | 1239 | 93.73 |
| AB4 | 72566 | 70795 | 252 | 6705 | 867 | 97.56 |
| AB5 | 63348 | 57419 | 252 | 6280 | 1202 | 90.64 |
| AB6 | 69409 | 67689 | 253 | 6164 | 694 | 97.52 |
| AFB1 | 61381 | 56718 | 252 | 5927 | 1063 | 92.4 |
| AFB2 | 75810 | 71781 | 252 | 5970 | 579 | 94.68 |
| AFB3 | 70259 | 68251 | 252 | 8722 | 1227 | 97.14 |
| AFB4 | 86235 | 80136 | 251 | 3811 | 1030 | 92.93 |
| AFB5 | 71141 | 67730 | 252 | 7777 | 835 | 95.2 |
| AFB6 | 78634 | 75879 | 252 | 6674 | 1045 | 96.5 |
| Total | 3741104 | 3547824 | 12088 | 237686 | 44834 | 4551.98 |
| Average | 77939.67 | 73913 | 251.83 | 4951.79 | 934.042 | 94.83 |
